# Comprehensive effects of Parkinson’s disease multimodal complex treatment (PD-MCT): a two-center prospective trial

**DOI:** 10.1007/s00415-026-13697-1

**Published:** 2026-02-18

**Authors:** Isabel Friedrich, Laura Gutschow, David Weise, Jost-Julian Rumpf, Joseph Classen, Christopher Fricke

**Affiliations:** 1 Department of Neurology, University of Leipzig Medical Centre, Liebigstrasse 20, 04103 Leipzig, Germany; 2 Department of Physical Therapy and Rehabilitation, Section Neurology, University Hospital Leipzig, Liebigstrasse 20, 04103 Leipzig, Germany; 3 Department of Neurology and Pain Therapy, Asklepios Fachklinikum Stadtroda, Bahnhofstrasse 1a, 07646 Stadtroda, Germany

**Keywords:** Parkinson’s disease, Inpatient treatment, Multimodal complex treatment, Motor symptoms, Quality of life

## Abstract

**Introduction:**

Parkinson’s disease (PD) is a progressive neurodegenerative disorder characterized by motor and non-motor symptoms that impair quality of life. Inpatient Parkinson’s disease Multimodal Complex Treatment (PD-MCT) integrates individualized medication adjustments with intensive multiprofessional therapies and improves symptoms and functional abilities. However, evidence remains limited regarding which symptom domains benefit most and which patient characteristics predict treatment response, particularly in advanced PD and with respect to cognitive status.

**Methods:**

This two-center, prospective cohort study included 53 adults with advanced idiopathic PD who underwent 14–21 days of inpatient PD-MCT at two centers. Inclusion criteria comprised a diagnosis of PD according to German and International Parkinson and Movement Disorder Society guidelines and the ability to walk at least 50 m with assistive devices. Patients with atypical parkinsonian syndromes or comorbidities severely affecting gait were excluded. Motor function, non-motor symptoms, cognition, and quality of life were assessed at baseline and before discharge. A 3-month follow-up evaluated symptom burden and continuation of supportive therapies. Primary outcomes were changes in motor symptom severity (ΔMDS-UPDRS-III), overall motor function (ΔMDS-UPDRS-II + III + IV), and quality of life (ΔPDQ-39).

**Results:**

PD-MCT significantly improved motor symptom severity (−5.2 ± 10.6), overall motor function (−10.6 ± 10.7), and quality of life (−9.3 ± 16.2). Additional benefits were observed in non-motor symptoms, cognition, mood, and functional measures. Greater baseline motor impairment and poorer quality of life predicted larger short-term improvements, while baseline cognition did not influence treatment effectiveness. Predictive modelling showed modest predictive value of baseline motor scores for changes in MDS-UPDRS-III.

**Conclusion:**

These findings confirm that PD-MCT provides clinically meaningful motor, non-motor, cognitive, and quality-of-life benefits in advanced PD, with strongest effects in patients with high baseline symptom burden.

## Introduction

Parkinson’s disease (PD) is among the most common neurodegenerative disorders, with incidence and prevalence rising with age [1, 2]. According to International Parkinson and Movement Disorder Society diagnostic criteria, Parkinson’s disease is characterized by the presence of bradykinesia along with at least one of the following cardinal motor signs: resting tremor or rigidity [3]. Beyond these motor signs, postural instability as well as a wide spectrum of non-motor symptoms (NMS) substantially impairs quality of life (QoL). NMS include hyposmia, REM sleep behavior disorder and other sleep disturbances, depression, autonomic dysfunction, and cognitive impairment progressing to dementia [4, 5]. As no disease-modifying therapy is currently available [6], treatment remains symptomatic. Alongside dopaminergic medication, exercise and supportive therapies are essential to preserve mobility, independence, and participation [7, 8]. With disease progression, motor fluctuations and dyskinesia often require complex drug adjustments or invasive interventions, such as continuous drug delivery or deep brain stimulation. Outpatient optimization of such complications is time-consuming and difficult to monitor, making inpatient Parkinson’s disease Multimodal Complex Treatment (PD-MCT) an attractive option, particularly in advanced stages or in patients with complex symptom burdens [9–14].

PD-MCT is delivered by a multiprofessional team of neurologists, nurses, and therapists specialized in movement disorders. Treatment goals are defined on admission, and medication adjustments are combined with intensive supportive therapies, such as physiotherapy, occupational, speech, sports, or music therapy [15]. The standardized inpatient stays range from short-term admissions (≥ 7 days, corresponding to German operation and procedure code OPS 8-97d.0) to extended interventions exceeding 21 days (OPS 8-97d.2) (OPS-2025).

Assessing the effectiveness and the long-term effects of PD-MCT has been the focus of recent clinical trials. Evidence from retrospective [11, 16] and prospective trials [9, 10, 17–20] indicates that PD-MCT improves motor and non-motor symptoms, functional mobility, and quality of life. Maintenance of improvement appears to depend strongly on continued physical activity, and improvements have been reported to gradually diminish within 6–12 months [16, 21, 22] raising the issue of whether and when benefits can persist after discharge. Minimal clinically important differences (MCIDs) have been established for key PD outcomes such as MDS-UPDRS subscores II, III and IV, and PDQ-39 score [23–27]. However, several PD-MCT studies have failed to demonstrate improvements of MCID-level changes in self-care, daily functioning (MDS-UPDRS-II), or motor complications (MDS-UPDRS-IV), raising the question of clinical relevance [11, 17, 18]. It is therefore essential to investigate whether measurable improvements are clinically significant and functionally relevant. Although PD-MCT allows some flexibility in individual therapeutic components, its highly structured design may lead to lesser benefit in some patients. To inform efforts toward personalizing PD-MCT, it is crucial to examine how treatment effectiveness depends on patient characteristics. Such analyses could facilitate the development of a more personalized therapy with greater efficacy and more targeted resource allocation. Indeed, whether particular patient characteristics are predictive of benefits from PD-MCT remains an open question. Finally, while prospective monocentric studies support the effectiveness of PD-MCT [9, 10, 17, 18, 28], robust multicenter data remain scarce.

In this exploratory study, we aimed to: (1) characterize domain-specific response patterns in motor, non-motor, and quality-of-life outcomes following PD-MCT in the first two-center cohort, providing novel insights beyond previous single-center studies and contributing additional evidence on real-world treatment responses, (2) identify potential predictors of individual responses using a predictive approach not previously applied in this context, and (3) examine the persistence of treatment effects across different domains at 3-month follow-up, highlighting patterns of lasting versus transient changes.

By combining a two-center design with an expanded multimodal assessment—including disease-specific and comprehensive cognitive tests, fine-motor tasks, and detailed balance measures—this study provides a more nuanced characterization of therapy effects especially in later-stage PD.

## Methods

### Trial design and participants

**Fig. 1:**
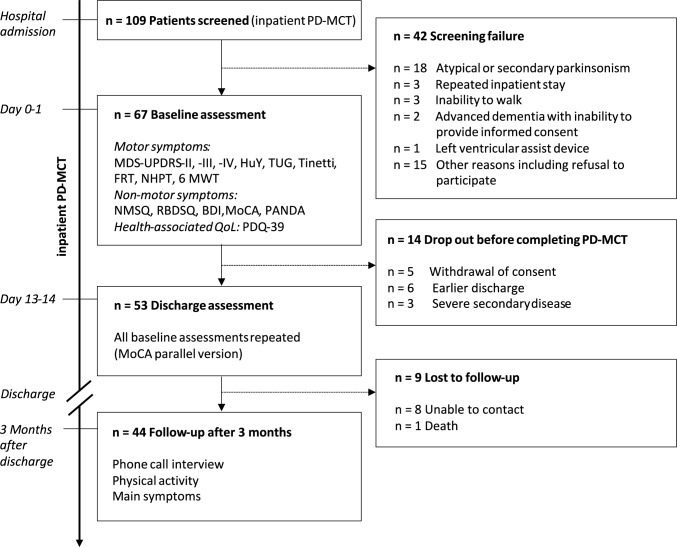
Trial design and measurements

The trial was designed as a two-center, prospective, open-label, non-randomized cohort trial. All trial participants underwent 14–21 days of inpatient PD-MCT. Recruitment took place between September 2022 and August 2024 at the Department of Neurology at the University of Leipzig Medical Centre, Germany, and the Department of Neurology and Pain Therapy at the Asklepios Clinic Stadtroda, Germany. Across both trial sites, a total of 109 patients were screened for eligibility, of whom 67 met the inclusion criteria and completed the baseline assessment (see Fig. 1). Prior to trial participation, all patients signed written informed consent for inclusion. The study was conducted in accordance with the ethical standards of the responsible ethics committees and with applicable national and institutional guidelines. Ethical approval was obtained from the Ethics Committee of the Faculty of Medicine, University of Leipzig (approval number: 215/22-ek; primary approval), and from the State Medical Association of Thuringia (registration number: 79148/2023/67). Experienced neurologists who were not involved in any trial-specific assessments carried out the selection of trial participants at the trial site Stadtroda. Inclusion criteria were (I) the diagnosis of sporadic or genetic PD based on the German Guidelines [29] and the International Parkinson and Movement Disorder Society (MDS) Clinical Diagnostic Criteria [3], (II) a minimum age of 18 years and (III) the ability to walk at least 50 m with assistive devices. Patients with atypical or secondary Parkinson’s syndrome were excluded from trial participation. Further exclusion criteria comprised conditions likely to influence gait due to secondary causes, including recent cerebral infarction with residual leg weakness (within 6 months), recent hip or knee arthroplasty (within 6 months), comorbid idiopathic or secondary hydrocephalus with manifest gait disturbance, severe peripheral arterial occlusive disease (Fontaine stage > IIB), and any lower extremity joint stiffness. In addition, severe clinical PNP, defined as the presence of distal pareses, sensory ataxia, and absent lower extremity reflexes, was an exclusion criterion; no patients met these criteria. Electrophysiological testing was not routinely performed. Pregnancy and breastfeeding were also exclusionary.

Each included patient received PD-MCT. The PD-MCT at both trial sites met the requirements published by the Federal Institute for Drugs and Medical Devices (BfArM) and stipulated in the Operation and Procedure Classification System (OPS) (corresponds to, e.g., CPT-Code, USA; OPCS-4, Office of Population Censuses and Surveys Classification of Interventions and Procedures. UK; CCAM, Classification Commune des Actes Médicaux, France). These included a weekly therapy duration of at least 7.5 h including at least 5 h individual therapy by at least three therapeutic areas (physiotherapy, occupational therapy, speech therapy, sports therapy, etc.) as well as multiprofessional team meetings at least once a week. According to the annual number of PD-MCT carried out at each trial site, trial sites can be classified as centers with regular to frequent treatment experience [30].

### Experimental procedures

In both centers, baseline examinations were conducted within 48 h (day 0 or 1) of hospital admission, with a second assessment on days 13 or 14, shortly before planned discharge. Between these visits, participants underwent PD-MCT according to standard clinical procedures. Demographic and medical history data were collected at baseline. Assessments included functional status (modified Barthel Index, mBI) and a multimodal evaluation of motor symptoms in the medication-ON state, covering self-reported daily function (MDS-UPDRS-II), overall motor symptom severity (MDS-UPDRS-III), motor complications (MDS-UPDRS-IV), balance, gait, and endurance (Timed Up and Go Test, TUG; Tinetti Gait and Balance Test; Functional Reach Test, FRT; 6-Minute Walking Test, 6MWT, patient-reported fall frequency within 6 months prior to admission), and fine-motor skills (Nine-Hole Peg Test, NHPT). Assessments also encompassed non-motor symptoms (Non-Motor Symptom Questionnaire, NMSQ; Beck Depression Inventory, BDI; REM Sleep Behavior Disorder Screening Questionnaire, RBDSQ); cognitive performance (Montreal Cognitive Assessment, MoCA; Parkinson’s Neuropsychometry Dementia Assessment, PANDA); and patient-reported quality of life (Parkinson’s Disease Questionnaire-39 items, PDQ-39). At 3 months post-discharge, participants were contacted for a structured telephone interview to assess symptom burden, engagement in physical activities, and continuation of supportive therapies. All assessments were performed by trained neurologists and therapists not involved in PD-MCT delivery or subsequent care. Participant numbers, assessment details, and trial flow are summarized in Fig. 1.

We additionally performed a quantitative assessment of gait and postural response, the results of which will be reported in another article.

### Outcome parameters

We derived three main outcome parameters for which we expected a significant improvement due to PD-MCT: *Change in motor symptom severity* was assessed as the pre-minus-post difference in MDS-UPDRS-III score in ON medication state (ΔMDS-UPDRS-III). An *overall motor sum score* was calculated by summing the pre-post changes in MDS-UPDRS-II, -III, and -IV (ΔMDS-UPDRS-II + III + IV). Finally, changes in *patient-reported quality of life* were captured by the pre-minus-post difference in PDQ-39 score (ΔPDQ-39). For ΔMDS-UPDRS-III and ΔPDQ-39 we defined *clinically meaningful changes* using prespecified, previously defined, thresholds [23, 24] classifying all patients as *responders*, *stable*, or *worsened* (ΔMDS-UPDRS-III—responders: < −3.25, worsened: > 3.25; ΔPDQ-39—responders: > 4.72, worsened < −4.72)*.*

We additionally explored treatment-dependent changes in supplementary scores related to fine-motor skills, cognitive abilities, and mood. Regarding cognition we assessed six cognitive subdomains derived from MoCA and PANDA (attention/working memory, verbal episodic memory, language, semantic fluency, visuospatial/executive function, and orientation).

### Statistical analysis

Given the exploratory, open-label design of this two-center cohort study, all analyses were hypothesis-generating. The primary null hypothesis stated that there are no systematic within-subject changes in ΔMDS-UPDRS-III, ΔMDS-UPDRS-II + III + IV, or ΔPDQ-39 beyond random variation, and that patients are not more likely to be classified as responders than as stable or worsened across outcome domains.

Statistical analyses were performed using *R* (version 4.5.1; R Foundation for Statistical Computing, Vienna, Austria) and *Matlab* (version 2024a, Mathworks, Natick, USA). Descriptive statistics were calculated for all baseline variables and are presented as mean ± standard deviation (SD) or median and interquartile range (IQR), depending on distributional properties. Group differences in baseline characteristics between trial sites and other categorical subgroups as well as pre–post-treatment differences were assessed using independent- or paired-sampled t tests for normal distributed continuous variables, and Wilcoxon tests for non-normally distributed continuous variables (Table 1). Normality of distributions for test selection was evaluated by Shapiro–Wilk test in combination with visual inspection of histograms and Q–Q plots. Differences between at least three independent groups were first analyzed using Kruskal–Wallis tests for continuous variables, because variables did not meet the assumptions of normality and homogeneity of variances required for parametric testing. When a significant overall group effect was observed, pairwise post hoc comparisons were performed using Dunn’s test with Bonferroni correction to identify specific group differences.

**Table 1:** Baseline characteristics

	Total cohort (*n* = 53)	Trial center Leipzig (*n* = 32)	Trial center Stadtroda (*n* = 21)	*p* value
Age, years	70.7 ± 7.5	69.2 ± 7.1	72.9 ± 7.6	0.089
Female, % (n)	45.3 (24)	40.6 (13)	52.4 (11)	0.576
Years since diagnosis, median (IQR)	11.0 (4.0–14.0)	11.5 (5.8–15.2)	10.0 (3.0–14.0)	0.247
Modif. BI, median (IQR)	90 (85–95)	90 (85–95)	90 (75–100)	0.719
LEDD, mg/day	825.1 ± 476.4	894.3 ± 532.7	719.6 ± 361.6	0.161
H&Y stage, median (IQR)	2.5 (2.0–3.0)	2.5 (2.0–3.0)	2.5 (2.0–3.0)	0.656
MDS-UPDRS-II	17.2 ± 6.5	18.2 ± 6.0	15.6 ± 7.2	0.214
MDS-UPDRS-III	35.6 ± 13.1	33.5 ± 11.5	38.7 ± 15.0	0.241
MDS-UPDRS-IV	5.1 ± 4.5	5.6 ± 4.7	4.3 ± 4.3	0.347
MoCA	23.2 ± 5.7	24.3 ± 4.2	21.5 ± 7.3	0.422
PANDA	19.6 ± 7.2	19.9 ± 7.1	19.2 ± 7.5	0.870
PDQ39	53.2 ± 18.9	54.3 ± 18.3	51.3 ± 20.5	0.615
BDI	11.8 ± 8.1	12.3 ± 8.0	10.9 ± 8.5	0.469
Tinetti test	19.7 ± 6.1	19.5 ± 5.7	19.9 ± 6.9	0.858
TUG	13.1 ± 7.1	12.7 ± 7.9	13.8 ± 5.9	0.206
6MWT	344.4 ± 145.4	371.4 ± 151.2	304.4 ± 129.7	0.094
NPHT dominant side	33.6 ± 22.9	35.4 ± 27.9	31.0 ± 11.8	0.842

Data are presented as mean ± SD unless otherwise indicated. Group differences in baseline characteristics between trial sites and other categorical subgroups were assessed using independent-samples t tests for normal distributed continuous variables, or Wilcoxon tests for non-normally distributed continuous variables. Significant differences were marked as **bold**

We evaluated associations between baseline characteristics and outcome parameters by calculating Pearson’s correlation coefficients for individual parameter pairs, in addition to employing linear regression models. The linear models incorporated a selection of baseline motor, cognitive, and functional metrics as predictors: baseline MDS-UPDRS-II, -III, and -IV, fall frequency, BDI, MoCA, PANDA, NMSQ, Tinetti Test, TUG, 6-MWT, patient age, Levodopa equivalent daily dose (LEDD), and disease duration. The outcome parameters detailed earlier were designated as response variables. To enhance the model’s interpretability while maximizing the informational content per variable and to avoid overfitting given the comparably large number of predictors, we utilized stepwise regression, reporting only the predictors that were kept as relevant for the final models. After outlining observable associations across the entire dataset, we used the selected predictors and retrained the regression model while employing fivefold cross-validation during model fitting to evaluate the predictive capabilities of the linear models. Training was repeated 20 times to yield stable classifiers. Explained variance, model significance, and the root-mean-square error (RMSE) were used to describe model performance. All statistical tests were two-tailed, and an alpha error < 0.05 was considered statistically significant. No adjustments for multiple comparisons were applied to exploratory correlation and regression analyses unless otherwise stated.

Exploratory causal mediation analysis was used to decompose the total effect of training specifics on outcome parameters into average direct effects (ADE) and average causal mediation effects (ACME) effects. ACME represents the effect mediated through a third variable, while ADE is the remaining direct effect. Estimates were obtained using the *R mediation package* with 5000 nonparametric bootstrap resamples for bias-corrected 95% confidence intervals. Continuous variables were standardized, and effects were considered significant if the confidence interval excluded zero.

## Results

### Demographics and baseline characteristics

From September 2022 to August 2024, we screened patients who underwent inpatient PD-MCT. 67 met the inclusion criteria, provided written informed consent, and completed the baseline assessment. Major reasons for exclusion were advanced PD with inability to walk at least 50 m even with assistive devices or diagnosis of atypical Parkinsonian syndromes. At discharge, 53 patients completed the second assessment. Reasons for dropout are summarized in Fig. 1. A *complete-case* analysis including 53 participants was performed to maintain the reliability of statistical inferences. Baseline characteristics are summarized in Table 1. Participants presented with advanced PD, with impaired postural responses and motor fluctuations in a substantial proportion of the cohort (median H&Y stage: 2.5, IQR: 2.0–3.0; MDS-UPDRS-IV: 5.1 ± 4.5, LEDD in mg/d: 825.1 ± 476.4). Despite a moderate mean motor symptom severity (MDS-UPDRS-III: 35.6 ± 13.1), functional independence was largely preserved (median mBI: 90, IQR: 85–95), but cognitive impairment was frequent, typically of mild-to-average severity (MoCA: 23.2 ± 5.7; PANDA: 19.6 ± 7.2). No significant differences regarding patient characteristics were observed between the two centers.

**Fig. 2:**
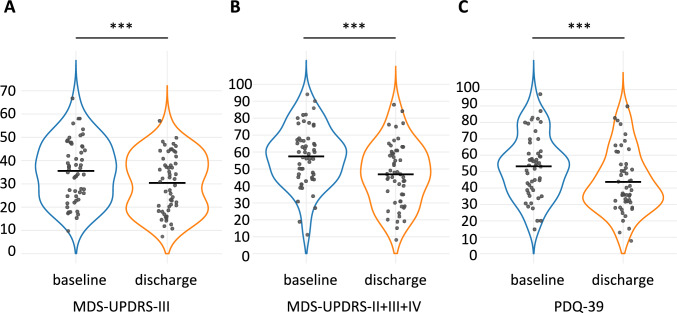
Violin plots illustrating the three primary outcomes: **A** change in motor symptom severity assessed by the MDS-UPDRS Part III in the medication-ON state, **B** change in the combined MDS-UPDRS sum score (Parts II + III + IV), and **C** change in patient-reported disease-specific quality of life assessed by the PDQ-39. Baseline assessments are shown in blue and discharge assessments in orange. Statistically significant differences (*p* < 0.001) are indicated by ***. Horizontal lines within the plots represent mean values

Correlation analyses of baseline motor, cognitive, mood, and functional metrics revealed multiple significant associations (Fig. 2A). Of note, motor symptom scores (MDS-UPDRS-II, -III, -IV) were strongly interrelated and associated with reduced functional independence (BI), impaired balance and decreased mobility (TUG, Tinetti), and poorer QoL (PDQ-39). Cognitive performance metrics (MoCA, PANDA) were closely interrelated, associated with better functional capacity (6MWT), and inversely related to motor symptom severity (MDS-UPDRS-III), while higher depressive symptoms (BDI) corresponded to greater non-motor symptom burden (NMSQ) and lower QoL (PDQ-39). Higher Levodopa equivalent daily dose (LEDD) was significantly associated with poorer Barthel Index (BI) scores (*r* = −0.325, *p* = 0.021), increased motor fluctuations (*r* = 0.377, *p* = 0.005), worse quality of life (PDQ39, *r* = 0.289, *p* = 0.042), and lower functional mobility (Functional Reach Test, *r* = −0.295, *p* = 0.032).

The occurrence of fall events was associated with a number of motor and non-motor symptoms. Based on self-reported fall frequency in the six months prior to admission [31], we categorized patients as nonfallers (no falls), single fallers (one fall), and recurrent fallers (two or more falls). Recurrent fallers exhibited a more advanced disease stage compared with single fallers and nonfallers (H&Y stage: recurrent fallers 2.9 ± 0.8, single fallers 2.9 ± 0.7, nonfallers 2.4 ± 0.5; Kruskal–Wallis *p* = 0.023; post hoc: recurrent vs. nonfallers *p* = 0.046), lower cognitive performance (MoCA: recurrent fallers 21.0 ± 6.1, single fallers 24.8 ± 6.9, nonfallers 24.4 ± 4.2; Kruskal–Wallis *p* = 0.021; post hoc: recurrent vs. single faller *p* = 0.024), and greater impairment in mobility and balance (TUG: recurrent fallers 15.2 ± 7.8, single fallers 15.4 ± 9.2, nonfallers 10.0 ± 4.6 s; Kruskal–Wallis *p* = 0.013; post hoc: recurrent vs. nonfaller *p* = 0.017; Tinetti: recurrent fallers 17.2 ± 7.3, single fallers 18.9 ± 5.8, nonfallers 22.3 ± 3.9; Kruskal–Wallis *p* = 0.022; post hoc: recurrent vs. nonfaller *p* = 0.020).

### Effectiveness of PD-MCT

**Fig. 3:**
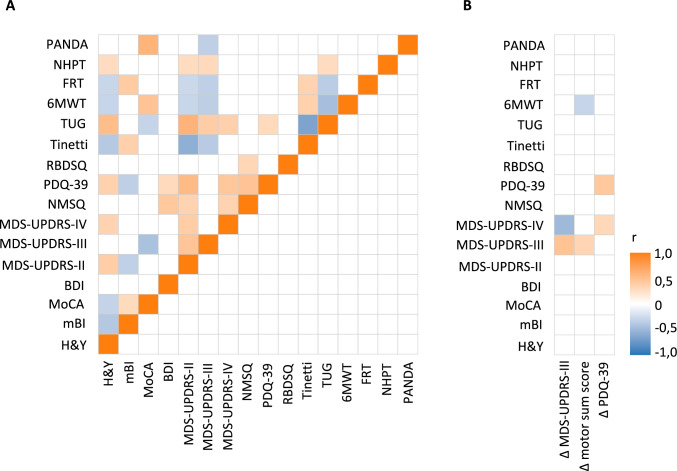
Correlation structure of baseline variables and outcome measures. Heatmaps depicting correlations at baseline (**A**) and between baseline measures and the three primary outcomes (**B**): change in motor symptom severity as measured by the MDS-UPDRS Part III in the medication-ON state, change in the combined MDS-UPDRS total score (Parts II + III + IV), and change in patient-reported disease-specific quality of life assessed by the PDQ-39. Statistically significant correlations (*p* < 0.050) are color-coded. Correlation coefficient (*r*) is provided for both heatmaps

PD-MCT led to significant changes across motor and non-motor domains, such as cognition and mood, and patient-reported quality of life (detailed description: Table 2, main outcome parameter: Fig. 3).

**Table 2:** Baseline and discharge assessments

	Baseline assessment	Discharge assessment	Effect size	*p* value
MDS-UPDRS sum score (II + III + IV)	57.4 ± 17.5	46.8 ± 18.5	0.733	**< 0.001**
LEDD in mg/d	825.1 ± 476.4	1082.2 ± 480.0	0.813	**< 0.001**
MDS-UPDRS-II	17.2 ± 6.7	13.4 ± 6.7	0.644	**< 0.001**
MDS-UPDRS-III	35.6 ± 13.1	30.4 ± 12.4	0.489	**< 0.001**
MDS-UPDRS-III subscore bradykinesia	18.3 ± 7.3	14.8 ± 7.2	0.531	**< 0.001**
MDS-UPDRS-IV	5.1 ± 4.5	2.7 ± 3.2	0.663	**< 0.001**
PDQ39 sum score	53.1 ± 19.1	43.8 ± 19.0	0.576	**< 0.001**
PDQ39 mobility	0.44 ± 0.24	0.36 ± 0.20	0.372	**0.009**
PDQ39 ADL	0.36 ± 0.23	0.31 ± 0.20	0.438	**0.002**
PDQ39 communication	0.30 ± 0.21	0.22 ± 0.18	0.441	**0.002**
PDQ39 body discomfort	0.36 ± 0.23	0.27 ± 0.23	0.399	**0.008**
PDQ39 emotional wellbeing	0.33 ± 0.19	0.29 ± 0.23	0.235	0.107
PDQ39 stigma	0.21 ± 0.25	0.17 ± 0.20	0.226	0.113
PDQ39 social support	0.15 ± 0.19	0.12 ± 0.17	0.215	0.132
H&Y	2.7 ± 0.7	2.6 ± 0.7	0.228	0.097
mBI	88.3 ± 11.7	90.8 ± 9.9	0.249	0.081
NMSQ	11.8 ± 3.9	10.0 ± 3.7	0.552	**< 0.001**
BDI	12.1 ± 8.0	10.7 ± 8.7	0.315	**0.031**
TUG	13.1 ± 7.2	11.4 ± 5.1	0.501	**< 0.001**
Tinneti Test	19.7 ± 6.1	20.4 ± 4.8	0.252	0.066
6MWT	344.5 ± 146.9	412.1 ± 133.3	0.666	**< 0.001**
FRT	26.3 ± 9.4	29.7 ± 10.1	0.375	**0.007**
NHPT dominant side	33.8 ± 23.1	28.5 ± 13.6	0.336	**0.015**
PANDA sum score	19.6 ± 7.3	21.6 ± 6.5	0.498	**< 0.001**
PANDA paired-associate memory	3.3 ± 1.9	4.0 ± 1.6	0.341	**0.014**
PANDA verbal fluency	3.8 ± 2.2	4.5 ± 1.9	0.334	**0.016**
PANDA visuoconstructive abilities	3.1 ± 1.5	3.7 ± 1.5	0.313	**0.024**
PANDA working memory	4.2 ± 1.7	4.3 ± 1.6	0.014	0.917
PANDA episodic long-term memory	4.2 ± 2.9	4.7 ± 2.4	0.223	0.107
MoCA	23.2 ± 5.7	23.4 ± 3.9	0.002	0.987

Data are presented as mean ± SD unless otherwise indicated pre–post-treatment differences were assessed using paired-samples t tests for normal distributed continuous variables, or Wilcoxon tests for non-normally distributed continuous variables. Significant differences were marked as **bold**

At baseline, gait and balance disturbances (*n* = 34), fine-motor impairments (*n* = 25), and pain (*n* = 13) were the most frequently reported symptoms.

Overall symptom burden (mean NRS of three main symptoms) decreased from 6.1 ± 1.8 at admission to 4.1 ± 2.0 at discharge (*p* < 0.001). With respect to *motor outcome*, we found that the overall motor sum score (MDS-UPDRS-II + III + IV) decreased by 10.6 ± 10.7 points (*p* < 0.001), driven by reductions in the MDS-UPDRS-III (− 5.2 ± 10.6, *p* < 0.001) and MDS-UPDRS-II (− 3.8 ± 5.5, *p* < 0.001). Stratifying responders with the MCID threshold, 32 patients (60.4%) showed clinically meaningful improvement in the MDS-UPDRS-III, 10 (18.9%) remained stable, and 11 (20.8%) worsened. Responders had lower baseline MDS-UPDRS-IV scores (responders: 3.1 ± 3.7, stable: 5.8 ± 4.7, worsened: 7.8 ± 5.1; Kruskal–Wallis *p* = 0.011; post hoc: responders vs. worsened *p* = 0.015) and LEDD was highest in the worsened subgroup (responders: 806 ± 450 mg, stable: 540 ± 453 mg, worsened: 1140 ± 419 mg; Kruskal–Wallis *p* = 0.008; post hoc: worsened vs. stable *p* = 0.008, worsened vs. responders *p* = 0.044). Patient’s reported *quality of life*, assessed by PDQ-39, improved by 9.3 ± 16.2 points (*p* < 0.001), with mobility, activities of daily living, communication, and bodily discomfort subdomains all showing significant gains (p ≤ 0.009). For PDQ-39, we classified 26 patients (49.1%) as responders, 20 (37.7%) as stable, and 7 (13.2%) as worsened, without them featuring significant baseline differences.

PD-MCT was associated with significant improvements in scores representing a variety of PD non-motor symptoms (NMSQ), as well as representing walking speed (TUG), balance (Tinneti, FRT), fine-motor skills (NHPT), total cognition including several cognitive subdomains (PANDA), as well as mood (BDI; Table 2).

In the telephone interview 3 months after treatment, we found a partial rebound of the overall symptom burden to 5.1 ± 1.7 (*p* = 0.007), yet symptoms remained significantly lower than at baseline (*p* < 0.001). This indicates a sustained clinically meaningful improvement. Among participants, 74.5% reported continuing supportive therapies (physiotherapy 66.6%, occupational therapy 27.5%, and speech therapy 23.6%), averaging 1.1 ± 1.0 h per week. Additionally, 43.1% engaged in other physical activities resulting in a total weekly activity—including supportive therapy—of 2.3 ± 2.5 h per week.

### Associations between baseline and outcome assessments and treatment outcome prediction

We examined associations between baseline measures and treatment response across three outcomes (ΔMDS-UPDRS-II + III + IV, ΔMDS-UPDRS-III, ΔPDQ-39), expressed as pre-to-post differences (Fig. 2B).

More severe baseline motor symptoms (higher MDS-UPDRS-III) were correlated to greater improvements in both overall motor sum score (ΔMDS-UPDRS-II + III + IV, *r* = 0.33, *p* = 0.016) and motor symptom severity (ΔMDS-UPDRS-III, *r* = 0.47, *p* < 0.001). Similarly, reduced baseline mobility (lower 6MWT) was associated with greater gains in overall motor sum score (ΔMDS-UPDRS-II + III + IV, *r* = −0.29, *p* = 0.035). Analogously, patients reporting poorer quality of life (higher PDQ-39) at baseline also experienced the greatest improvements in this domain (ΔPDQ-39, *r* = 0.43, *p* = 0.002). In contrast, more pronounced motor complications at baseline (higher MDS-UPDRS-IV) were associated with smaller gains in motor symptom severity (ΔMDS-UPDRS-III, *r* = −0.49, *p* < 0.001) but higher achieved improvements in quality of life (ΔPDQ-39, *r* = 0.30, *p* = 0.033). Treatment-related changes in motor function and quality of life were independent of baseline performance in cognitive tests including subdomains (MoCA and PANDA, |*r*|≤ 0.18, *p* ≥ 0.170). There were no significant correlations between LEDD at baseline nor of the change in LEDD to neither outcome score (|*r*|≤ 0.269, *p* ≥ 0.0612).

**Fig. 4:**
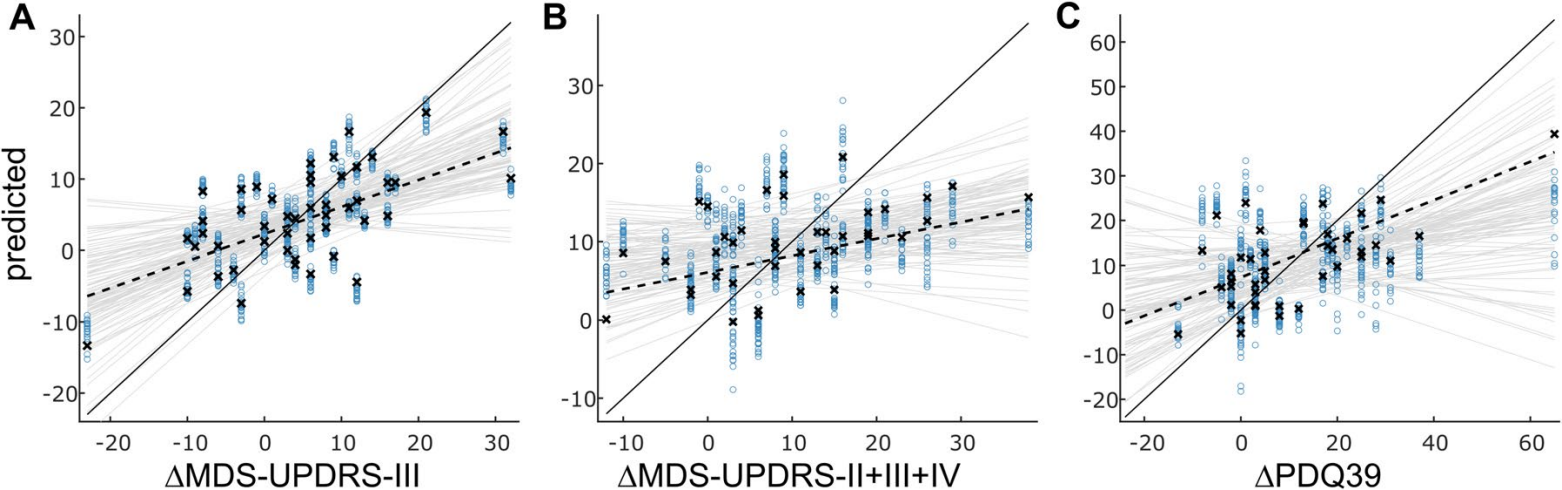
Linear regression models of the outcome parameters ΔMDS-UPDRS-III, ΔMDS-UPDRS-II + III + IV, and ΔPDQ39. In all three plots, the crosses and black dashed line represent the fit over the whole dataset, while the black diagonal line represents the optimal fit. The light blue circles and light gray lines represent individual cross-validation model fits. Only the model for the ΔMDS-UPDRS-III (**A**) had meaningful predictive capabilities, in the figure indicated by a similar individual out-of-sample predictions during cross-validation compared with the modeling over the whole dataset. The other models (**B** and **C**)—though significant for the whole dataset—tended to overfit and deviate strongly from the optimal (**B**) as well as whole-dataset-model (**C**). Note, in particular, the wide scattering of individual predicted points as well as of the line slopes in B and C compared to A

Expanding upon the observed individual correlations between baseline clinical measures and therapy responses, we evaluated the feasibility of integrating these parameters into a predictive model for individual outcomes using stepwise linear regression with baseline parameters as regressors and ΔMDS-UPDRS-II + III + IV, ΔMDS-UPDRS-III, and ΔPDQ-39 as response variables as detailed in Table 3 and depicted in Fig. 4. Very similar to the correlation analysis, we found that that baseline MDS-UPDRS-III was positively (*p* = 0.002), while baseline MDS-UPDRS-IV was negatively (*p* < 0.001) associated with the improvement in MDS-UPDRS-III following therapy (ΔMDS-UPDRS-III, model *R*^2^ = 0.402, RMSE = 8.203, *p* < 0.001). With respect to the overall motor outcome (ΔMDS-UPDRS-II + III + IV, model *R*^2^ = 0.244, RMSE = 9.777, *p* = 0.039), we found a significant association with baseline walking endurance (6MWT, *p* = 0.013), whereas baseline MDS-UPDRS-IV showed only a trend toward significance (*p* = 0.074). Additionally, there was now a trend for the PANDA baseline score (*p* = 0.061) hinting at some additional benefit of higher baseline cognitive functioning regarding motor outcome. The BDI was non-significant but retained in the model (*p* = 0.295). Quality-of-life outcome (model *R*^2^ = 0.395, RMSE = 12.095, *p* < 0.001) mostly depended on its own baseline value (*p* < 0.001) as in the correlation analysis and additionally on a faster baseline TUG (*p* = 0.020).

**Table 3:** Linear regression models for outcome parameters

Stepwise linear regression models	β-estimate [95%-CI]	*p* value
Outcome: ΔMDS-UPDRS-II + III + IV		**0.039**
MDS-UPDRS-IV	−2.97 [−6.13, 0.19]	0.074
6MWT	−4.26 [−7.45, −1.08]	**0.013**
PANDA	3.32 [−0.04, 6.69]	0.061
BDI	1.72 [−1.45, 4.90]	0.295
Outcome: ΔMDS-UPDRS-III		**< 0.001**
MDS-UPDRS-III	3.90 [1.59, 6.21]	**0.002**
MDS-UPDRS-IV	−4.60 [−6.97, −2.22]	**< 0.001**
Outcome: ΔPDQ-39		**< 0.001**
MDS-UPDRS-IV	0.93 [−3.65, 5.50]	0.694
TUG	−5.06 [−9.12, −0.99]	**0.020**
PDQ39	9.50 [4.87, 14.14]	**< 0.001**
BDI	−2.08 [−6.35, 2.18]	0.344

Baseline parameters kept in the model by stepwise regression are detailed with their β-estimates with 95%-confidence interval (CI) and *p* values. Significant differences were marked as **bold**

We explored the predictive capabilities of these models using fivefold cross-validation during model fitting with the parameters selected by the stepwise regression procedure as regressors. We found that ΔMDS-UPDRS-III could be predicted from the selected regressors with about 30% of variance of the outcome metric explained from baseline MDS-UPDRS-III and -IV values (ΔMDS-UPDRS-III, *R*^2^ = 0.299 ± 0.074, median 0.324). The model tended to underestimate extreme changes in both directions (Fig. 4A). We tested the hypothesis that the model could be improved upon by adding PANDA and BDI as interaction terms given a possible influence of cognition and mood on outcome parameters. We did not find relevant improvements; instead, the regressor now tended to overfit (ΔMDS-UPDRS-III, *R*^2^ = −0.087 ± 0.202, median 0.124). For neither the total motor score nor the health-related quality of life, we found a meaningful predictive linear model, neither using the whole set of parameters from Table 3, including PANDA and BDI nor using only significant regressors (average *R*^2^ ≤ 0.068, Fig. 4B + C).

### Exploratory analysis of mediation effects of therapy settings on motor outcome

We conducted exploratory causal mediation analyses to assess the role of therapy settings in motor recovery. These analyses indicated that overall therapy length, time spent in group sessions, and individual therapy time did not significantly mediate improvements in any primary outcome (ΔMDS-UPDRS-II + III + IV, ΔMDS-UPDRS-III, ΔPDQ-39; all *p* ≥ 0.314), except for a trend-level mediation of individual therapy time on motor symptom improvement (ΔMDS-UPDRS-III: ACME = −0.085, 95%-CI [−0.210, 0.005], *p* = 0.072), while direct effects of baseline predictors on post-treatment outcomes remained significant (all ADE *p* < 0.001).

## Discussion

This trial demonstrated in a first two-center cohort that PD-MCT administered to patients with PD and moderate-to-severe symptom burden significantly reduced motor symptom severity, improved overall motor function, and enhanced patient-reported quality of life. Importantly, improvements persisted, at least in part, at 3-month follow-up, indicating that benefits extend beyond the immediate post-intervention period. Our results indicate that baseline MDS-UPDRS-III and IV measures provide a clear and relevant predictive contribution to motor symptom severity at discharge, adding meaningful value beyond previous approaches.

Although cross-country comparability is limited by heterogeneous PD-MCT definitions, the standardized OPS framework in Germany ensures consistent minimum requirements, enabling meaningful benchmarking across centers. Baseline characteristics of our cohort align well with prior reports [14, 15, 22]. However, our cohort included patients with a comparatively longer disease duration than most previous studies, allowing the investigation of PD-MCT effects across a broader range of PD stages. Our findings confirm and substantially extend the previous evidence from monocentric, largely retrospective studies, which were often limited to short-term outcomes assessed immediately after termination of the intervention. By employing a prospective, two-center design under real-world conditions, this study appears to provide substantial new evidence that PD-MCT produces meaningful and durable benefits also in patients with advanced PD, supporting its value as a structured, yet adaptable therapeutic approach. Improvements were found across multiple domains, including functional ability (MDS-UPDRS-II), motor symptom severity (MDS-UPDRS-III), therapy-related motor complications (MDS-UPDRS-IV), and disease-specific quality of life (PDQ-39), matching or even exceeding previous findings [9–11, 17, 18], and surpassing established MCID thresholds [23, 24, 27, 32]. By applying highest published MCID thresholds where possible, we ensured that reported responder rates reflect meaningful improvements that concur with patients’ perceptions, rather than functionally meaningless improvements that are indistinguishable from random fluctuations.

A key strength of this trial lies in its comprehensive, multidimensional characterization of patients, integrating assessments of motor and non-motor symptom burden, cognition, and patient-reported quality of life. By combining examiner-based and patient-reported measures, we captured the broad impact of PD-MCT beyond single endpoints. Multidimensional assessments did not identify patients with isolated symptoms of overwhelming significance. Instead, we found measures at baseline to be strongly interrelated. This included associations between cognitive and motor performance, non-motor symptom burden, including mood symptoms, and quality of life. For example, we observed that patients with recurrent falls, a surrogate marker of advanced disease and postural instability, exhibited more pronounced impairments across multiple additional domains, illustrating how clusters of deficits capture heightened vulnerability [31]. Cognitive impairment affected approximately two-thirds of the cohort, alongside prominent motor symptoms and fluctuations, reflecting the substantial multisystemic nature of neurodegeneration of advanced PD.

The broad improvements across multiple domains likely reflect synergistic effects of intensive physical training, optimized pharmacotherapy, and active engagement with therapeutic staff. Reviews consistently demonstrate that motor exercises may enhance strength [33, 34], coordination, and balance [35] while also supporting functional independence and daily activities [7, 36]. Cognitive engagement during therapy may further facilitate executive function and attention, partly through increased dopaminergic availability and effects neuroplasticity [37, 38], thereby indirectly improving motor performance. In parallel, medication adjustments may reduce fluctuations and symptom severity, enabling fuller participation in therapeutic activities.

Patients with greater baseline motor impairments achieved substantial absolute motor improvements, demonstrating that PD-MCT can induce clinically relevant benefits even in more severely affected individuals. Several mechanisms likely contribute to the comparatively larger improvements in these patients: Stronger neurophysiological activation during training may provide greater benefit to those with lower baseline motor function. Likewise, pharmacological optimization may yield more pronounced effects in patients suffering from greater motor fluctuations or higher baseline symptom burden. These considerations are supported by the observation that out-of-sample prediction was possible only for ΔMDS-UPDRS-III from its baseline value (MDS-UPDRS-III) and MDS-UPDRS-IV which likely represents too low dopaminergic stimulation at baseline.

While baseline measures were informative predictors of improvement in motor symptom severity (ΔMDS-UPDRS-III), individual prediction of overall motor performance (ΔMDS-UPDRS-II + III + IV) or quality of life (ΔPDQ-39) remained unreliable. Several pathophysiological factors likely contribute, including the heterogeneous symptomatology of PD, involvement of different neural systems, and the multifactorial determinants of non-motor and quality-of-life outcomes. Predictive power is also affected by methodological limitations due to the small sample size and the broad scope of the outcome measures provoking overfitting. Consequently, a high proportion of outcome variance remained unexplained, likely reflecting unmeasured biological, cognitive, and psychosocial factors (e.g., motivation, adherence, and cognitive reserve). The models’ tendencies to underestimate extreme responses likely reflect both the limited sample size and the inherent variability of patient trajectories. However, our findings suggest that while baseline severity can provide guidance, PD-MCT currently cannot be withheld from an individual patient based on negative model prediction, even when patient’s characteristics are known in great detail. From a clinician’s point of view, these findings support the notion that offering PD-MCT to patients with a high degree of motor symptom load with low dyskinesia scores—thus must likely patients with insufficiently low dopaminergic stimulation—will also most likely result in an improvement of motor symptom severity. Importantly, neither baseline nor change in LEDD was significantly correlated to the improvement in any of the outcome parameters, suggesting that even though dopaminergic stimulation was significantly increased on average, this most likely represents only one component of the efficacy of PD-MCT. Thus, given the overall rather unpredictable but clearly positive expected development of the quality of life, our findings also support offering PD-MCT broadly to all patients with limitations in this respect rather than restricting therapy based on baseline motor symptom severity alone.

At 3 months post-discharge, patients showed substantial retention of benefits, consistent with the previous reports [16, 17]. However, the previous work has also demonstrated that initial gains may later wane without continued support [22]. Because our assessment was terminated at 3 months, we cannot determine the persistence of treatment effect in the longer term. We consider it likely that structured post-treatment programs or home-based interventions are required to maintain achieved improvements.

This study was conducted as an exploratory investigation to examine variability in treatment responses across motor, non-motor, and quality-of-life domains in patients receiving inpatient Multimodal Complex Treatment (PD-MCT), and to identify potential predictors of response.

Several important limitations must be acknowledged. An open-label design and absence of a control group severely limit causal inference and preclude formal testing of a null hypothesis. The open-label format was chosen deliberately to allow detailed, domain-specific observation in this exploratory setting, rather than to formally test treatment efficacy. Randomization or a control group was ethically and practically challenging, as all included patients had a clinical indication for inpatient PD-MCT due to complex symptom burden not manageable in an outpatient setting. Patient and investigator expectations may have influenced outcomes, and placebo effects cannot be excluded. These may have contributed substantially to the observed improvements, particularly given the intensive, multimodal, and inpatient nature of the intervention. Placebo responses are known to be pronounced in Parkinson’s disease and can induce clinically relevant motor improvements mediated by dopaminergic mechanisms [39]. In addition, random fluctuations may have influenced the results. While all assessments were conducted in the ON medication state and at comparable times of day pre- and post-treatment to reduce such variability, the observed changes should be interpreted cautiously, reflecting overall treatment-associated responses in a real-world inpatient setting rather than definitive treatment-specific efficacy.

Further, therapy content and formats—including the balance of individual and group sessions—were not fully standardized across both centers, although this did not appear to influence outcomes. We cannot entirely exclude selection bias, as in one center, only a subset of all patients admitted for PD-MCT could be screened. However, since baseline characteristics and outcomes did not differ significantly between centers, incomplete recruitment has unlikely led to skewed results. The 3-month follow-up relied on telephone assessments only, limiting the temporal scope of the present study. An additional limitation might be seen in the fact that it was based on only two centers. Therefore, the trial results may still not generalize to different national or international settings. The modest sample size, on the other hand, constrained the feasibility and potential complexity of predictive models and drove overfitting for too large parameter spaces warranting the use of stepwise regressions. In this context, larger cohorts might allow the detection of more subtle patterns but most likely with little individual predictive meaning. Nevertheless, the consistency of baseline characteristics and outcomes across centers supports the feasibility of pooling data in future analyses to increase statistical power and enable robust evaluation of PD-MCT effectiveness, including subgroup analyses.

## Conclusion

In this first prospective two-center study, PD-MCT produced clinically meaningful improvements across motor symptoms and motor complications, functional mobility, fine-motor skills, non-motor burden—including cognition and mood—and quality of life. Patients with severe baseline motor symptom load benefitted particularly well from PD-MCT in this domain but predictive modeling indicated that these relationships were of limited value and other relevant outcome parameters (e.g., quality of life) were not predictable at all but nevertheless can be expected to improve due to PD-MCT. Although symptom improvements persisted at 3-month follow-up, the reduction in symptom burden was only partially sustained. These results underscore PD-MCT’s broad, functionally significant effects and emphasize the necessity of structured post-treatment follow-up to maximize and sustain patient benefit.

## Data Availability

The datasets generated and analyzed during the current trial are available from the corresponding author on reasonable request.

## Abbreviations

6MWT: 6-Minute Walking Test
ACME: Average causal mediation effect
ADE: Average direct effect
AIC: Akaike information criterion
ANN: Artificial neural network
BDI: Beck Depression Inventory
BfArM: Federal Institute for Drugs and Medical Devices (Germany)
FRT: Functional Reach Test
H&Y: Hoehn and Yahr stage
IQR: Interquartile range
LEDD: Levodopa equivalent daily dose
MCID: Minimal clinically important differences
MDS-UPDRS: International Parkinson and Movement Disorder Society—Unified Parkinson’s Disease Rating Scale
mBI: Modified Barthel Index
MoCA: Montreal Cognitive Assessment
NHPT: Nine-Hole Peg Test
NMSQ: Non-Motor Symptom Questionnaire
OPS: Operation and Procedure Classification System (Germany)
PANDA: Parkinson’s Neuropsychometry Dementia Assessment
PD: Parkinson’s disease
PD-MCT: Parkinson’s disease multimodal complex treatment
PDQ-39: Parkinson’s Disease Questionnaire-39 items
QoL: Quality of life
RBDSQ: REM Sleep Behavior Disorder Screening Questionnaire
REM: Rapid eye movement
RMSE: Root-mean-square error
SD: Standard deviation
SVM: Support vector machine
TUG: Timed Up and Go Test
